# Chemical composition and resistance-modifying effect of the essential oil of *Lantana camara* Linn

**DOI:** 10.4103/0973-1296.62890

**Published:** 2010-05-05

**Authors:** Erlânio O. Sousa, Natálya F. Silva, Fabiola F. G. Rodrigues, Adriana R. Campos, Sidney G. Lima, José Galberto M. Costa

**Affiliations:** *Programa de Pós-Graduação em Bioprospecção Molecular, Departamento de Química Biológica, Laboratório de Pesquisa de Produtos Naturais, Universidade Regional do Cariri, Rua Cel. Antônio Luiz 1161, Pimenta, 63105-000, Crato-CE, Brazil*; 1 *Vice-Reitoria de Pesquisa e Pós-Graduação, Universidade de Fortaleza, Av. Washington Soares 1321, Edson Queiroz, 60811-905, Fortaleza-CE, Brazil*; 2 *Departamento de Química, Universidade Federal do Piauí, Campus Universitário Ministro Petrônio Portella, 64049-550, Bairro Ininga, Teresina-PI, Brasil*

**Keywords:** *Lantana camara* Linn, essential oil, aminoglycosides, antibacterial and modulatory activities

## Abstract

In this work, the chemical constituents, antibacterial and modulatory activities of the essential oil of *Lantana camara* Linn were studied. The essential oil was extracted from the leaves of *L. camara* by hydrodistillation method using Clevenger's apparatus and its chemical constituents were separated and identified by GC-MS, and the relative content of each constituent was determined by area normalization. Among the 25 identified components, bicyclogermacrene (19.42%), isocaryophyllene (16.70%), valecene (12.94%) and germacrene D (12.34%) were the main constituents. The oil was examined to antibacterial and modulatory activities against the multiresistant strains of *Escherichia coli* and *Staphylococcus aureus* by microdilution test. The results show an inhibitory activity to *E. coli* (MIC 512 μg/ml) and *S. aureus* (MIC 256 μg/ml). The synergism of the essential oil and aminoglycosides was verified too, with significant reduction of MICs (7 ×, 1250-5 μg/ml) against *E. coli*. It is suggested that the essential oil of *Lantana camara* Linn could be used as a source of plant-derived natural products with resistance-modifying activity.

## INTRODUCTION

The emergence and spread of antimicrobial resistance justifies the high investment in the search of new drugs from vegetable species, in order to combat multiresistant microorganisms.[1–4] Among the bacterial genera able to develop changes in their sensitivity to antimicrobials, *Staphylococcus* species have been recognized as having a worrying increase in antimicrobial resistance.[5,6]

Despite causing different kinds of intoxications, *S. aureus* has been the most common etiological agent of festering infections that attack different tissues and/or organs (e.g. furuncle, carbuncle, abscess, myocarditis, endocarditis, pneumonia, meningitis, bacterial arthritis).[7,8]

*Escherichia coli* is a part of the intestinal microflora of most animals and humans and it is commonly associated with non complicated respiratory infections. Many reports have shown that *E. coli* has a tendency to be resistant to a range of antibiotics, mainly the β-lactamase producers.[9]

Five strains of *E. coli*, enterophatogenic *E. coli* (EPEC), enteroaggregative *E. coli* (EAEC), enterotoxigenic *E. coli* (ETEC), enteroinvasive *E. coli* (EIEC) and Shiga toxin-producing *E. coli* (STEC), have been associated with human diseases. These *E. coli* categories are known to cause diarrhea and are responsible for diverse intestinal and extraintestinal diseases.[9]

Lantana is a genus of about 150 species of perennial flowering plants popularly used as antirheumatic, stimulant, antibacterial, biologic control and as ornamental plant.[10,11]

*Lantana camara* Linn (camará) is a shrub that belongs to Verbenaceae family. It is a native of America and Africa and has been cultivated as an ornamental plant in other countries. In Brazil, *Lantana camara* is found throughout al regions.[11,12] Different parts of the plant, mainly the leaves, have been used in the treatment of scratching, stomachache, rheumatism, wound healing, biliary fever, toothache, bronquitis, antiseptic and other affections.[11,13] In Brazil, the leaves are used to treat rheumatism and pulmonary diseases.[14]

The chemical study of essential oil of *L. camara* leaves revealed the presence of a high amount of sesquiterpenes and this oil remarkably inhibited the growth of most tested bacteria and fungi, *Pseudomonas aeruginosa, Aspergillus niger, Fusarium solani* and *Candida albicans* appearing as the most sensitive.[13]

The oil is reported to possess insecticidal and repellent activities toward bees, mosquitoes and cattle flies too. Dua *et al*.[10] showed that the essential oil of flowers has repellent effect against *Aedes* mosquitoes. This study was undertaken to screen the phytochemical composition and antimicrobial modulatory activity of *Lantana camara* Linn leaves essential oil from Cariri Cearense, Brazilian Northeast.

## MATERIALS AND METHODS

### Plant material

Leaves of *Lantana camara* Linn were collected in March, 2009, from the Small Aromatic and Medicinal Plants Garden of the Natural Products Research Laboratory (LPPN) at University Regional do Cariri (URCA), Crato of county, Ceará state, Brazil.

A voucher specimen was sent to the Herbarium Caririense Dárdano de Andrade Lima - HCDAL Department of Biological Sciences (URCA), which is deposited on the registration N° 1619.

### Obtention of essential oil

Samples of fresh leaves (500 g) were triturated and submitted to hydrodistillation process, in a Clevenger-type apparatus for 2 h. The collected essential oil of *Lantana camara* (EOLc) was subsequently dried by anhydrous sodium sulfate (Na_2_ SO_4_), and stored under refrigeration at < 4 °C until be analyzed and tested.

### Analysis of essential oil

Analysis by CG/MS of the essential oil was carried out on a Hewlett-Packard Model 5971 GC/MS using a non-polar DB-1 fused silica capillary column (30 m × 0.25 mm i.d., 0.25 μm film thickness); carrier gas helium, flow rate 0.8 ml/min and with split mode. The injector temperature and detector temperature were 250°C and 200°C, respectively. The column temperature was programmed from 35°C to 180°C at 4°C/min and then 180°C to 250°C at 10°C/min. Mass spectra were recorded from 30 to 450 *m/z*. Individual components were identified by matching their 70 eV mass spectra with those of the spectrometer data base using the Wiley L-built library and two other computer libraries MS searches using retention indices as a pre-selection routine,[15,16] as well as by visual comparison of the fragmentation pattern with those reported in the literature.[17]

### Investigation of antibacterial activity and minimal inhibitory concentration (MIC)

The antibacterial activity of the essential oil was investigated employing a microdilution method, recommended by NCCLS M7-A6.[18] In the assay, two multiresistant strains, *Escherichia coli* (27) from sputum and *Staphylococcus aureus* (358) from surgical wound, obtained from clinical material were used.

Brain hear infusion broth (BHI 3.8%) was used for bacterial growth (24 h, 35 ± 2°C). The inoculum was an overnight culture of each bacterial species in BHI broth diluted in the same media to a final concentration of approximately 1 × 10^8^ CFU/ml (0.5 nephelometric turbidity units - McFarland scale). After this, the suspension was diluted to 1 × 10^6^ CFU/ ml in 10% BHI. About 100 μl of each dilution was distributed in 96-well plates plus essential oils, achieving 5 × 10^5^ CFU/ml as final concentration of the inoculum.

Essential oil was dissolved in distilled water and dimethyl sulfoxide (DMSO) to a concentration of 1024 μg/ml. Further serial dilutions were performed by the addition of BHI broth to reach a final concentration in the range of 8–512 μg/ml. All experiments were performed in triplicate and the microdilution trays were incubated at 35 ± 2°C for 24 h. Antibacterial activity was detected using a colorimetric method by adding 25 μl of resauzurin staining (0.01%) aqueous solution in each well at the end of the incubation period. The minimal inhibitory concentration (MIC) was defined as the lowest essential oil concentration able to inhibit the bacteria growth, as indicated by resauzurin staining (bacteria died cells are not able to change the staining color by visual observation – blue to red).

### Modulatory activity evaluation

To evaluate the EOLc as a modulator of antibiotic resistance, the MICs of aminoglycosides (neomycin, canamycin, amikacin and gentamicin) against the analyzed strains were determined in the presence or absence of EOLm using the microdilution test. Subinhibitory concentrations (MIC 1/8) in 10% BHI were used.

The antibiotics solutions (5000 μg/ml) were prepared in distillated water for use the same day. A total of 100 μl of the antibiotic solution, using use serial dilutions (1:2), was added to the wells containing 10% BHI and the diluted bacterial suspension (1:10). Microplates were incubed for 24 h at room temperature and the antibacterial activity was determined as described before.

## RESULTS AND DISCUSSION

The essential oil obtained by hydrodistillation gave a yield of 0.12% (w/w). GC/MS analysis permitted the identification and quantification of twenty-five constituents (99.75%), with predominance of sesquiterpenes (56%) and a little amount of monoterpenes (44%) [Table 1]. These results are consistent with the previous reports that show a large percentage of sesquiterpenes in *Lantana* especies essential oils.[19,20] Bicyclogermacrene (19.42%), isocaryophyllene (16.70%), valecene (12.94%) and germacrene D (12.34%) were the main constituents identified.

**Table 1:** Chemical components of *Lantana camara* fresh leaves essential oil

Components	IR^a^ sam.	IR^b^ lit.	(%)
Sabinene	971	971	4.78
*ß*-pinene	973	978	0.25
*ß*-myrcene	979	979	0.39
*ρ*-cymene	1020	1024	1.37
*Z*-*ß*-ocimene	1028	1026	0.94
*E*-*ß*-ocimene	1045	1045	1.02
*y*-terpinene	1046	1048	0.62
Terpinolene	1082	1086	0.83
cis-*p*-menth-2-en-1-ol	1103	1103	5.17
4-terpineol	1175	1175	0.59
Azulenol	1290	1292	5.56
α-elemene	1330	1331	1.59
α-copaene	1366	1376	0.42
ß-elemene	1385	1393	1.16
*E*-*ß*-caryophyllene	1400	1404	2.91
Isocaryophyllene	1409	1409	16.70
Alloaromadrene	1441	1443	2.29
*y*-elemene	1442	1434	1.59
α-humulene	1448	1455	2.07
germacrene D	1473	1474	12.34
Bicyclogermacrene	1490	1491	19.42
Valecene	1500	1496	12.94
*y*-cadinene	1514	1513	0,49
Spathulenol	1572	1576	2.58
caryophyllene oxide	1571	1569	1.73
Total identified			99.97
Monoterpernes			44
Sesquiterpenes			56

a Relative retention indices experimental: n-alkanes were used as reference points in the calculation of relative retention indices

b Relative retention indices (literature values)

Caryophyllene isomers are the common constituents in *Lantana* species, and until now, germacrene D and bicyclogermacrene are components that appear in essential oils from *Lantana* species. It has been shown recently that the *L. camara* essential oil from the south of China is characterized by the high percentage of these sesquiterpenes.[21]

The EOLc antimicrobial properties against two bacteria strains by using microdilution assay for *in vitro* susceptibility testing were investigated. An inhibitory activity, clinically relevant, was verified against *E. coli* (MIC 512 μg/ml) and *S. aureus* (MIC 256 μg/ml), Table 2. These results are consistent with that of the previous studies by Deena and Thoppil[13] where they verified the antibacterial potential from *L. camara* against gram-positives strains default and mainly gram-negatives.

**Table 2:** Minimal inhibitory concentration (MIC) values of *Lantana camara* fresh leaves essential oil

Strains	MIC (μg/ml)	Resistance profile
*E. coli* (27)	512	Ast, Ax, Amp, Ami, Amox, Ca, Cfr, Cf, Caz, Cip, Chl, Im, Kan, Szt, Tet, Tob.
*S. aureus* (358)	256	Oxa, Gen, Tob, Ami, Kan, Sis, Neo, Para, But, Net.

In an antibacterial screening using *Lantana achyranthiofolia* essential oil, an effective antibacterial activity against a multiresistant *Staphylococcus epidermidis* strain and *Vibrio cholerae* strains was verified.[22] This result indicates the strong antimicrobial potential of the *Lantana* species essential oil against multiresistant bacteria strains.

Table 3 shows the EOLc inteference on aminoglycosides activities (MIC 1/8). MICs reduction for all antibiotics used here was observed when EOLc was added to the growth medium. The most expressive effect was the potentiation of amikacin on *E. coli* by EOLc, with seven-fold MIC reduction (1250-5 μg/ml).

**Table 3:** Minimal inhibitory concentration (MIC) values of aminoglycosídes in the absence and presence of *Lantana camara* fresh leaves essential oil

Antibiotics	*E. coli* (27)		*S. aureus* (358)	
		
	MIC	EOLc(64 μg/ml)	MIC	EOLc(32 μg/ml)
Neomycin	625	5	1250	40
Amikacin	1250	5	1250	40
Kanamycin	1250	10	512	78
Gentamicin	312	2	2500	625

EOLc: essential oil of *L. camara*

According to Oliveira *et al*.,[23] the essential oil antimicrobial modifying effect is dependent on the antibiotic, essential oil tested and analyzed bacteria strain types.

Few works report the antibiotic potentializing effect by essential oils when in combination with classical antibiotics. Individual compounds of essential oils from *Melaleuca leucodendron* and *Ocimum gratissimum* presented synergism with several antibiotics by direct contact.[24] As far as we know, there is no report about the potentiation of aminoglycoside effects by *L. camara* or other *Lantana* specie.

Several reports indicate different antibiotic combinations assayed *in vitro* and applied in the clinics, but combinations of natural products and synthetic drugs are not reported. The results showed here are indicative that *L. camara* can play a role as a source of natural products acting as an antibiotic resistance modifier that can be used against multiresistant bacteria.
